# Modeling and Predicting Hemorrhagic Fever with Renal Syndrome Trends Based on Meteorological Factors in Hu County, China

**DOI:** 10.1371/journal.pone.0123166

**Published:** 2015-04-13

**Authors:** Dan Xiao, Kejian Wu, Xin Tan, Jing Le, Haitao Li, Yongping Yan, Zhikai Xu

**Affiliations:** 1 Department of Epidemiology, School of Public Health, Fourth Military Medical University, Xi’an, China; 2 Department of Mathematics and Physics, School of Biomedical and Engineering, Fourth Military Medical University, Xi’an, China; 3 Hu County Center for Disease Control and Prevention, Xi’an, China; 4 Hu County Meteorological Bureau, Xi’an, China; 5 Department of Microbiology, Fourth Military Medical University, Xi’an, China; Queensland University of Technology, AUSTRALIA

## Abstract

**Background:**

Hu County is a serious hemorrhagic fever with renal syndrome (HFRS) epidemic area, with notable fluctuation of the HFRS epidemic in recent years. This study aimed to explore the optimal model for HFRS epidemic prediction in Hu.

**Methods:**

Three models were constructed and compared, including a generalized linear model (GLM), a generalized additive model (GAM), and a principal components regression model (PCRM). The fitting and predictive adjusted R^2^ of each model were calculated. Ljung-Box Q tests for fitted and predicted residuals of each model were conducted. The study period was stratified into before (1971–1993) and after (1994–2012) vaccine implementation epochs to avoid the confounding factor of vaccination.

**Results:**

The autocorrelation of fitted and predicted residuals of the GAM in the two epochs were not significant (Ljung-Box Q test, P>.05). The adjusted R^2^ for the predictive abilities of the GLM, GAM, and PCRM were 0.752, 0.799, and 0.665 in the early epoch, and 0.669, 0.756, and 0.574 in the recent epoch. The adjusted R^2^ values of the three models were lower in the early epoch than in the recent epoch.

**Conclusions:**

GAM is superior to GLM and PCRM for monthly HFRS case number prediction in Hu County. A shift in model reliability coincident with vaccination implementation demonstrates the importance of vaccination in HFRS control and prevention.

## Introduction

Hemorrhagic fever with renal syndrome (HFRS) is a rodent-borne zoonosis caused by hantaviruses. It is characterized by varying degrees of bleeding diathesis, hypertension, and renal failure [1]. The overwhelming majority of reported cases of HFRS in Asia have occurred in China and the annual HFRS incidence of China has ranked No.1 in the world since 2000 [2]. The incidence of HFRS in Hu County ranked third of all counties in China [3]. Both Hantaan viruses (carried by *Apodemus agrarius* mice that thrive in the wild) and Seoul viruses (carried by *Rattus norvegicus* rats that thrive in residential areas) have been detected in Hu County, though Hantaan virus infections are more prevalent.

The incidence of HFRS in China has decreased significantly since 1984 owing to improved housing conditions, improved hygiene, and human migration from rural areas to cities [4]. However, the incidence of HFRS in Hu County has shown large fluctuations in the same period. It decreased greatly from 300.57/100,000 people in 1984 to 9.53/100,000 in 2005, and then increased to 48.46/100,000 in 2010 [5]. These incidence rates are 33.89 times, 5.96 times, and 68.25 times the rates of China as a whole in these sample years, respectively. The cause underlying the re-emergence of HFRS in Hu County after 2005 is not known. Elucidating the dynamic tendencies of the HFRS epidemic in Hu County will be critical for enabling China to allocate medical health resources effectively and to develop an appropriate plan for the prevention and control of the disease in HFRS re-emergent areas.

The epidemiologic profile of HFRS is influenced by numerous environmental and social factors, including meteorological factors [6, 7], rodent density [6], and vaccination [8]. Meteorological factors affect the behavior and survival of rodents, and thus also influence rodent density and the likelihood of people having contact with rodent excrement. Accurate rodent density data are difficult to obtain because densities are estimated based on the numbers of rats caught by traps in fields and around houses. These catch numbers are influenced by many factors and catch frequency can be irregular.

Researchers have attempted to predict HFRS epidemics based on meteorological factors and obtained evidence supporting the notion that meteorological factors are the main factors influencing HFRS epidemic risk [9–13]. Although these studies have shown good predictive capacities, there has been inconsistency between models and regions in terms of predictor variables and coefficients, leaving it unclear which model is most appropriate for HFRS epidemic prediction. This variability may due, at least in part, to the fact that the meteorological factors affecting epidemics are themselves influenced by many other factors, such as elevation (relative to sea level), vaccination compliance, and which rat species live in a region. Therefore, a prediction model constructed according to data from one area would not be expected to be universally applicable. In order to predict HFRS epidemics with confidence, it is necessary to construct a prediction model according to the relationship between HFRS epidemics and meteorological factors within each specific area.

Several types of mathematical models have been used to predict HFRS epidemics, including the generalized linear model (GLM) [10–12], seasonal autoregressive integrated moving average model (SARIMAM) [9], and autoregressive integrated moving average model (ARIMAM) [14, 15]. The prior ARIMAMs were based purely on data describing HFRS cases in prior months as predictor variables, without consideration of other potential influencing factors. The prior GLMs and SARIMAM were based on the hypothesis that HFRS cases and meteorological factors are in linear or logarithmic relationships. However, case-factor relationships are not confined to a fixed function. Therefore, some studies have attempted to describe these unfixed relationships using a generalized additive model (GAM) and a principal components regression model (PCRM) [16–18]. The GAM is a multiple regression model where smooth functions are used instead of pre-specifying forms for explanatory variables [19]. It provides a flexible model with which to explore the relationship between response and explanatory variables. The PCRM is a multiple regression model that involves the use of the principal components extracted from explanatory variables as predictor variables. It removes multi-colinearity between explanatory variables by dimension reduction. However, data on the accuracy of HFRS epidemic prediction using a GAM or PCRM are still scarce. Therefore, it remains unclear which model type would be better for HFRS epidemic prediction, which hinders HFRS control and prevention.

This study aimed to explore the optimal model for HFRS epidemic prediction in Hu County based on meteorological factors. To obtain accurate and comparable results, we constructed and compared a GLM, a PCRM, and a GAM, three frequently-used and well-regarded model types.

## Materials and Methods

### Ethics statement

This study was approved by the Ethics Committee of Fourth Military Medical University (Grant No.: 2013026). All data analyzed in this study were anonymized. All patient records were anonymized and de-identified prior to analysis.

### Data collection and management

Monthly records of HFRS cases in Hu County from1971 through 2012 were obtained from the Hu Center for Disease Control and Prevention (CDC). HFRS is a national class B notifiable communicable disease in China, and Hu County is a monitor sentinels for HFRS [20]. All hospitals and clinics in Hu County are obliged to report HFRS cases to the Hu CDC through the National Infectious Disease Reporting System within 24 hours [21]. Before 1982, HFRS was diagnosed according to the national standard clinical criteria [22]. After 1982, HFRS was diagnosed first in hospitals and clinics and then confirmed by laboratory tests at the Hu CDC. Only a few cases in which sudden death occurred (3 cases in this study) were not confirmed in the Hu CDC laboratory. Annual population data for Hu County during the 1971–2012 period were determined based on information from the Hu Bureau of Statistics. Population was estimated from annual household registration records maintained by the local police departments. Monthly meteorological data for Hu County during the same period, including mean temperature (MT), mean maximum temperature (MaxT), mean minimum temperature (MinT), accumulative rainfall (R), and mean relative humidity (H), were obtained from the Hu Meteorological Bureau. Mean meteorological values were calculated by averaging data across the 18 meteorological stations in Hu County.

### Cut-off of study period

An HFRS vaccination program has been in place in Hu County since 1994 and has contributed to the area’s decreased HFRS incidence [8]. In order to avoid the influence of vaccination as a confounding factor, the study period was stratified into the epochs before (1971–1993) and after (1994–2012) implementation of the vaccine program. All data analyses and model constructions were conducted using data in each these two time periods.

### Cross-correlation analysis and autocorrelation analysis

A cross-correlation analysis between monthly HFRS cases and meteorological index sequences were conducted with a lag time of six months to enable detection of the dominant meteorological factors influencing HFRS infections. Cross-correlations could be identified when the cross-correlation coefficient (CCF) was more than double the standard error (SE). The autocorrelation analysis of monthly HFRS cases was performed to explore the relationship between HFRS infections in consecutive months with the aim of accounting for short-term temporal persistence and annual trends. Autocorrelations could be identified if the autocorrelation coefficient (AC) was more than double the standard error (SE). Those variables that correlated significantly with the number of HFRS cases were selected as preliminary predictor variables.

### Multi-collinearity analysis

The variance inflation factor (VIF), tolerance (T), and Spearman’s rank correlation coefficient were calculated to examine the degree of multi-collinearity among the preliminary predictor variables identified in the cross-correlation analysis. Multi-collinearity was identified when the VIF was greater than 10.

### Model construction

We constructed and compared three models using meteorological factors as explanatory variables. Model 1 was a GLM with a Poisson distribution and a log link performed after adjustment for autocorrelation, seasonality, and lag effects, including meteorological variables that were selected from the preliminary predictor variables by the stepwise least square method, and HFRS cases in previous months. Model 2 was a GAM employing the same response variables as model 1. For the GAM, predictor variables with smooth functions (including meteorological variables) were selected from the preliminary predictor variables by the backfitting method, and HFRS cases in previous months. The generalized cross-validation criterion was used to estimate the smoothing parameter in the GAM. Model 3 was a PCRM that examined monthly HFRS cases against predictor variables, including principal components that were extracted from the preliminary predictor variables and HFRS case numbers from previous months. A 10-fold cross-validation analysis was employed in the PCRM fitting process to determine the number of principal components that should be included in the model.

The three models were constructed as follows:

$$\begin{array}{l} \text{Model}\;1:\;{\hat{y}}_{t}=\alpha_{1}+\sum_{i=1}^{m}\sum_{k=0}^{p}\beta_{i}x_{i\left(t-k\right)}+\sum_{j=1}^{n}\gamma_{j}y_{t-j}\\ \text{Model}\;2:\;{\hat{y}}_{t}=\alpha_{2}+\sum_{i=1}^{m}\sum_{k=0}^{p}s_{i}\left(x_{i\left(t-k\right)}\right)+\sum_{j=1}^{n}s_{j}\left(y_{t-j}\right)\\ \text{Model}\;3:\;y_{t}=\alpha_{3}+\sum_{i=1}^{q}\omega_{i}Z_{i}+\sum_{j=1}^{n}\gamma_{j}y_{t-j} \end{array}$$

Both *s*_*i*_(·) and *s*_*j*_(·) are smooth functions of the predictor variables in Model 2; they are fitted using a cubic regression spline. The smoothing parameters are determined using restricted maximum likelihood. The variable *y*_*t*_ is the number of HFRS cases in time *t*, *ŷ*_*t*_ is the expected value of *y*_*t*_, *x*_*i*(*t*−*k*)_ represents the meteorological factors, and *Z*_*i*_ represents the principal components. The terms *α*_1_, *α*_2_, and *α*_3_ are constants. In these three models, the terms containing *x*_*i*(*t*−*k*)_ denote the effect of meteorological factors, the terms containing *y*_*t*−*j*_ describe the effects of auto regression, and the term $\sum_{i=1}^{q}\omega_{i}Z_{i}$ represents the effect of the principal components. The parameters *m*, *p*, *n*, and *q* represent the number of meteorological variables, the maximum lagged months of meteorological variables, the maximum lagged months of HFRS cases, and the number of principal components, respectively. The terms *β*_*i*_, *γ*_*i*_, and *ω*_*i*_ are coefficients for each of the variables.

The three models were fit with data from the first 80% of the timeline within each of the two study epochs (1971–1988 and 1994–2008), and then we predicted numbers of HFRS cases for the remaining 20% of each of the epochs using concurrent meteorological data.

### Model evaluation

The actual, fitted, and predicted values of monthly HFRS cases during each epoch of the three models were plotted with different colored lines. Fitting and predictive adjusted *R* ^2^ values were calculated, and Ljung-Box Q tests for fitted and predicted residuals were conducted to compare the fit and predictive ability of the three models. The adjusted *R* ^2^ were calculated as follows:

$$\text{Adjusted}\;R^{2}=1-\left(1-R^{2}\right)\times \frac{n-1}{n-p+1}$$

where *n* is the number of months included, *p* is the number of response variables in the model, and *R* ^2^ is the square of the correlation coefficient between fitted or predicted values and actual values. All of these analyses were conducted using R Project 3.0.2 (R Development Core Team, 2012).

## Results

### Descriptive analysis

**Fig 1:**
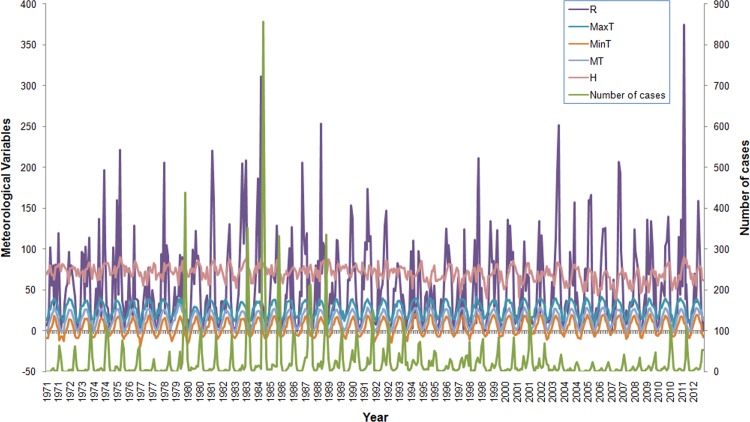
Temporal distribution of monthly HFRS cases and meteorological variables in Hu County, China, 1971–2012. There was obvious seasonality of HFRS epidemic in the study period, with peaks being observed in summer (June–July) and winter (October–December). The annual incidence fluctuated less than 10 cases per 100,000 people in 2005 to more than 300 in 1984.

There were 12,714 HFRS cases reported in Hu County between 1971 and 2012. The annual average incidence was 59.96 per 100,000, with the annual incidence ranging from 9.53/100,000 in 2005 (nadir) to 300.57/100,000 in 1984 (zenith). A clear seasonality phenomenon became apparent, with peaks occurring in summer and winter. The smaller of the two annual peaks occurred in summer between June and July, accounting for 9.88% of all the HFRS cases (1,256 cases). The larger peak occurred in winter between October and December and accounted for 75.49% of all HFRS cases (9,598 cases). The monthly MT, MaxT, MinT, R, and H ranged from -3.7°C to 33.3°C, from 5.3°C to 41.9°C, from -19.0°C to 20.5°C, from 0 mm to 374.9 mm, and from 39% to 90%, respectively. (Fig 1)

### Cross-correlation between monthly HFRS cases and meteorological factors

The number of HFRS cases reported each month correlated significantly with meteorological factors from the current and the previous month in both epochs (Table 1). The following variables correlated significantly with the monthly numbers of HFRS cases and thus were used as the preliminary predictor variables of the three models: MT_0_, MT_1_, MT_2_, MT_3_, MT_4_, MT_6_, MaxT_0_, MaxT_1_, MaxT_2_, MaxT_3_, MaxT_4_, MinT_0_, MinT_1_, MinT_2_, MinT_3_, MinT_4_, R_1_, R_2_, R_3_, R_4_, H_3_, H_4_ for the 1971–1993 epoch; and MT_1_, MT_2_, MT_3_, MT_4_, MT_5_, MaxT_1_, MaxT_2_, MaxT_3_, MaxT_4_, MinT_1_, MinT_2_, MinT_3_, MinT_4_, MinT_5_, R_1_, R_2_, R_3_, R_4_, R_5_, H_0_, H_1_, H_5_, H_6_ for the 1994–2012 epoch.

**Table 1:** Cross correlation between monthly hemorrhagic fever with renal syndrome cases and meteorological factors in Hu County, China during the 1971–1993 and 1994–2012 time periods.

lag	1971–1993										1994–2012									
	MT		MaxT		MinT		R		H		MT		MaxT		MinT		R		H	
	CCF	SE	CCF	SE	CCF	SE	CCF	SE	CCF	SE	CCF	SE	CCF	SE	CCF	SE	CCF	SE	CCF	SE
**-6**	0.202	0.067	0.208	0.067	0.151	0.067	0.101	0.067	-0.044	0.067	0.09	0.073	0.125	0.073	0.031	0.073	-0.11	0.073	-0.208	0.073
**-5**	0.361	0.067	0.344	0.067	0.336	0.067	0.223	0.067	-0.145	0.067	0.302	0.073	0.3	0.073	0.279	0.073	0.039	0.073	-0.223	0.073
**-4**	0.419	0.067	0.366	0.067	0.456	0.067	0.367	0.067	0.072	0.067	0.446	0.073	0.419	0.073	0.452	0.073	0.246	0.073	-0.033	0.073
**-3**	0.387	0.067	0.335	0.067	0.431	0.067	0.285	0.067	0.220	0.067	0.475	0.073	0.416	0.073	0.495	0.073	0.279	0.073	0.216	0.073
**-2**	0.235	0.067	0.186	0.067	0.270	0.067	0.452	0.067	0.370	0.067	0.368	0.073	0.357	0.073	0.39	0.073	0.265	0.073	0.382	0.073
**-1**	0.051	0.067	0.019	0.067	0.050	0.067	0.148	0.067	0.269	0.067	0.167	0.073	0.154	0.073	0.177	0.073	0.151	0.073	0.385	0.073
**0**	-0.171	0.067	-0.187	0.067	-0.176	0.067	-0.130	0.067	0.134	0.067	-0.09	0.073	-0.112	0.073	-0.082	0.073	-0.062	0.073	0.274	0.073
**1**	-0.368	0.067	-0.389	0.067	-0.337	0.067	-0.249	0.067	-0.006	0.067	-0.331	0.073	-0.373	0.073	-0.28	0.073	-0.244	0.073	0.202	0.073
**2**	-0.423	0.067	-0.448	0.067	-0.383	0.067	-0.262	0.067	-0.121	0.067	-0.471	0.073	-0.506	0.073	-0.415	0.073	-0.299	0.073	0.126	0.073
**3**	-0.364	0.067	-0.375	0.067	-0.336	0.067	-0.243	0.067	-0.211	0.067	-0.447	0.073	-0.435	0.073	-0.442	0.073	-0.309	0.073	-0.039	0.073
**4**	-0.221	0.067	-0.211	0.067	-0.212	0.067	-0.170	0.067	-0.135	0.067	-0.325	0.073	-0.277	0.073	-0.362	0.073	-0.289	0.073	-0.136	0.073
**5**	-0.025	0.067	-0.001	0.067	-0.039	0.067	-0.055	0.067	-0.074	0.067	-0.154	0.073	-0.069	0.073	-0.217	0.073	-0.246	0.073	-0.17	0.073
**6**	0.155	0.067	0.166	0.067	0.124	0.067	0.058	0.067	-0.055	0.067	0.056	0.073	0.098	0.073	0.013	0.073	-0.049	0.073	-0.151	0.073

CCF: cross correlation coefficient.

SE: standard error criterion.

Lag: the number of lagged months. Lag = 0 means the HFRS cases and meteorological factors occur in the same month.

The cross correlation could be identified if the CCF was greater than two times the SE

### Autocorrelation of monthly HFRS cases

**Fig 2:**
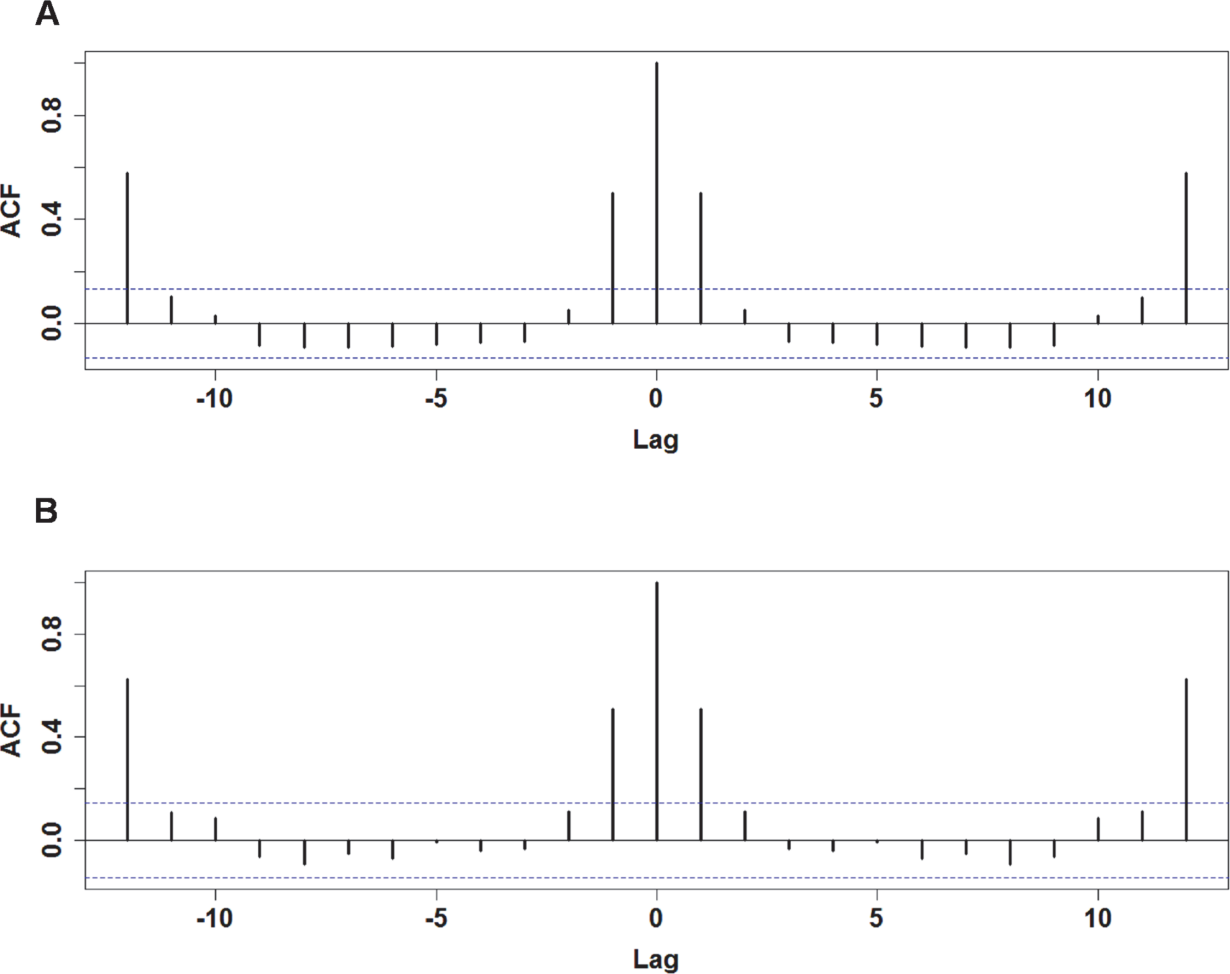
Autocorrelation of monthly HFRS cases. The pre-vaccination (1971–1993) and post-vaccination (1994–2012) autocorrelation coefficients are shown in A and B, respectively. The x-axis represents the number of lagged months, and y-axis shows the autocorrelation coefficient. The dotted lines denote the upper and lower levels of 2*SE.

The monthly numbers of HFRS cases correlated significantly with the numbers of cases in the first and twelfth lagged months in both epochs, pointing to a clear short-term temporal persistence effect and yearly trend (Fig 2). The variables N_1_ (no. cases in the immediately previous month) and N_12_ (no. cases in the month 12 months prior) were thus selected for inclusion as predictor variables in the models.

### Multi-collinearity among meteorological variables

There were 17 variables with variance inflation factors greater than 10 and corresponding tolerance values less than 0.1 in the early epoch, and 14 such variables in the recent epoch (Table 2). There were 193 pairs of variables that correlated with each other during the early epoch (Table 3, *P* < .05 or *P* < .01), and 226 pairs of variables that did so during the recent epoch (Table 4, *P* < .05 or *P* < .01). These results indicate that multi-collinearity of the preliminary predictor variables can be observed in this study.

**Table 2:** Collinearity diagnostics of preliminary predictor variables during the 1971–1993 and 1994–2012 epochs.

1971–1993			1994–2012		
Variable	VIF	Tolerance	Variable	VIF	Tolerance
**R**_1_	2.089	0.479	**R**_1_	2.396	0.417
**R**_2_	1.916	0.522	**R**_2_	2.492	0.401
**R**_3_	2.418	0.414	**R**_3_	2.374	0.421
**R**_4_	2.418	0.414	**R**_4_	2.420	0.413
**MaxT**_0_	17.811	0.056	**R**_5_	2.685	0.372
**MaxT**_1_	16.998	0.059	**MaxT**_1_	25.397	0.039
**MaxT**_2_	18.457	0.054	**MaxT**_2_	25.861	0.039
**MaxT**_3_	17.274	0.058	**MaxT**_3_	24.316	0.041
**MaxT**_4_	17.448	0.057	**MaxT**_4_	25.866	0.039
**MaxT**_6_	16.310	0.061	**MinT**_1_	46.152	0.022
**MinT**_0_	17.200	0.058	**MinT**_2_	47.272	0.021
**MinT**_1_	17.781	0.056	**MinT**_3_	44.870	0.022
**MinT**_2_	18.741	0.053	**MinT**_4_	48.965	0.020
**MinT**_3_	18.032	0.055	**MinT**_5_	44.363	0.023
**MinT**_4_	19.531	0.051	**MT**_1_	122.684	0.008
**MT**_0_	43.834	0.023	**MT**_2_	122.022	0.008
**MT**_1_	47.679	0.021	**MT**_3_	118.865	0.008
**MT**_2_	46.404	0.022	**MT**_4_	119.947	0.008
**MT**_3_	48.927	0.020	**MT**_5_	64.779	0.015
**MT**_4_	43.474	0.023	**H**_0_	2.148	0.466
**MT**_6_	32.013	0.031	**H**_1_	2.944	0.340
**H**_0_	2.189	0.457	**H**_5_	2.730	0.366
**H**_3_	2.712	0.369	**H**_6_	2.039	0.490
**H**_4_	2.454	0.408			

VIF: variance inflation factors for individual variables.

Tolerances: 1/VIF

**Table 3:** Spearman’s rank correlation coefficient between the preliminary predictor variables during the 1971–1993 epoch.

	MT_0_	MT_1_	MT_2_	MT_3_	MT_4_	MT_6_	MaxT_0_	MaxT_1_	MaxT_2_	MaxT_3_	MaxT_4_
**MT**_0_	1.00										
**MT**_1_	0.83**	1.00									
**MT**_2_	0.47**	0.83**	1.00								
**MT**_3_	0.00	0.47**	0.83**	1.00							
**MT**_4_	-0.47**	0.01	0.48**	0.83**	1.00						
**MT**_6_	-0.81**	-0.46**	0.01	0.48**	0.83**	1.00					
**MaxT**_0_	0.96**	0.77**	0.41**	-0.07	-0.52**	-0.83**	1.00				
**MaxT**_1_	0.84**	0.96**	0.77**	0.41**	-0.07	-0.52**	0.80**	1.00			
**MaxT**_2_	0.52**	0.84**	0.96**	0.77**	0.41**	-0.06	0.48**	0.81**	1.00		
**MaxT**_3_	0.06	0.52**	0.84**	0.96**	0.77**	0.41**	0.02	0.48**	0.80**	1.00	
**MaxT**_4_	-0.40**	0.07	0.53**	0.84**	0.96**	0.77**	-0.43**	0.02	0.48**	0.81**	1.00
**MinT**_0_	0.96**	0.84**	0.50**	0.03	-0.44**	-0.79**	0.92**	0.85**	0.54**	0.10	-0.37**
**MinT**_1_	0.79**	0.96**	0.84**	0.50**	0.04	-0.44**	0.73**	0.92**	0.85**	0.54**	0.11
**MinT**_2_	0.41**	0.79**	0.96**	0.84**	0.50**	0.04	0.36**	0.72**	0.92**	0.85**	0.55**
**MinT**_3_	-0.06	0.41**	0.79**	0.96**	0.84**	0.50**	-0.12	0.35**	0.72**	0.92**	0.85**
**MinT**_4_	-0.52**	-0.06	0.42**	0.79**	0.96**	0.84**	-0.56**	-0.11	0.36**	0.72**	0.92**
**R**_1_	0.58**	0.67**	0.65**	0.38**	0.03	-0.36**	0.54**	0.65**	0.66**	0.40**	0.09
**R**_2_	0.32**	0.58**	0.67**	0.65**	0.39**	0.03	0.26**	0.54**	0.65**	0.66**	0.41**
**R**_3_	-0.40	0.32**	0.59**	0.67**	0.66**	0.39**	-0.09	0.26**	0.54**	0.65**	0.66**
**R**_4_	-0.39**	-0.04	0.32**	0.59**	0.68**	0.66**	-0.43**	-0.09	0.26**	0.54**	0.66**
**H**_3_	-0.45**	-0.36**	-0.18**	0.02	0.33**	0.45**	-0.45**	-0.37**	-0.20**	-0.05	0.28**
**H**_4_	-0.44**	-0.44**	-0.36**	-0.17**	0.03	0.33**	-0.43**	-0.45**	-0.37**	-0.20**	-0.04
	**MinT**_0_	**MinT**_1_	**MinT**_2_	**MinT**_3_	**MinT**_4_	**R**_1_	**R**_2_	**R**_3_	**R**_4_	**H**_3_	**H**_4_
**MT**_0_											
**MT**_1_											
**MT**_2_											
**MT**_3_											
**MT**_4_											
**MT**_6_											
**MaxT**_0_											
**MaxT**_1_											
**MaxT**_2_											
**MaxT**_3_											
**MaxT**_4_											
**MinT**_0_	1.00										
**MinT**_1_	0.82**	1.00									
**MinT**_2_	0.46**	0.82**	1.00								
**MinT**_3_	-0.01	0.46**	0.82**	1.00							
**MinT**_4_	-0.47**	-0.01	0.47**	0.82**	1.00						
**R**_1_	0.60**	0.72**	0.64**	0.36**	-0.01	1.00					
**R**_2_	0.37**	0.60**	0.72**	0.64**	0.36**	0.50**	1.00				
**R**_3_	-0.01	0.37**	0.61**	0.72**	0.64**	0.25**	0.51**	1.00			
**R**_4_	-0.34**	-0.01	0.37**	0.61**	0.72**	-0.10	0.25**	0.51**	1.00		
**H**_3_	-0.45**	-0.33**	-0.14*	0.13*	0.37**	-0.21**	-0.06	0.46**	0.34**	1.00	
**H**_4_	-0.42**	-0.45**	-0.33**	-0.13*	0.13*	-0.46**	-0.20**	-0.05	0.46**	0.31**	1.00

*, *P* < 0.05.

**, *P* < 0.01

**Table 4:** Spearman’s rank correlation coefficient between the preliminary predictor variables during the 1994–2012 epoch.

	MT_1_	MT_2_	MT_3_	MT_4_	MT_5_	MaxT_1_	MaxT_2_	MaxT_3_	MaxT_4_	MinT_1_	MinT_2_	
**MT**_1_	1.00											
**MT**_2_	0.84**	1.00										
**MT**_3_	0.48**	0.84**	1.00									
**MT**_4_	0.01	0.48**	0.84**	1.00								
**MT**_5_	-0.46**	0.01	0.47**	0.84**	1.00							
**MaxT**_1_	0.96**	0.77**	0.40**	-0.08	-0.54**	1.00						
**MaxT**_2_	0.85**	0.96**	0.78**	0.39**	-0.08	0.80**	1.00					
**MaxT**_3_	0.54**	0.85**	0.97**	0.78**	0.40**	0.47**	0.80**	1.00				
**MaxT**_4_	0.09	0.54**	0.85**	0.97**	0.78**	-0.00	0.46**	0.80**	1.00			
**MinT**_1_	0.98**	0.86**	0.54**	0.08	-0.40**	0.93**	0.87**	0.59**	0.16*	1.00		
**MinT**_2_	0.79**	0.98**	0.87**	0.54**	0.08	0.72**	0.92**	0.88**	0.59**	0.83**	1.00	
**MinT**_3_	0.41**	0.79**	0.98**	0.87**	0.54**	0.32**	0.72**	0.92**	0.87**	0.47**	0.83**	
**MinT**_4_	-0.07	0.40**	0.78**	0.98**	0.87**	-0.17*	0.32**	0.72**	0.93**	-0.01	0.47**	
**MinT**_5_	-0.53**	-0.08	0.40**	0.79**	0.98**	-0.60**	-0.16*	0.32**	0.72**	-0.47**	0.00	
**R**_1_	0.66**	0.70**	0.52**	0.20**	-0.20**	0.62**	0.72**	0.55**	0.25**	0.70**	0.69**	
**R**_2_	0.51**	0.65**	0.70**	0.52**	0.20**	0.42**	0.61**	0.72**	0.55**	0.55**	0.69**	
**R**_3_	0.18**	0.51**	0.65**	0.69**	0.51**	0.09	0.42**	0.61**	0.71**	0.23**	0.55**	
**R**_4_	-0.20**	0.17**	0.51**	0.65**	0.69**	-0.27**	0.09	0.42**	0.61**	-0.13*	0.23**	
**R**_5_	-0.50**	-0.20**	0.17*	0.51**	0.65**	-0.54**	-0.27**	0.10	0.43**	-0.45**	-0.13	
**H**_0_	0.35**	0.55**	0.57**	0.46**	0.23**	0.31**	0.52**	0.58**	0.52**	0.41**	0.57**	
**H**_1_	0.02	0.36**	0.56**	0.57**	0.46**	-0.07	0.31**	0.52**	0.58**	0.13	0.41**	
**H**_5_	-0.53**	-0.58**	-0.49**	-0.26**	0.00	-0.51**	-0.59**	-0.52**	-0.32**	-0.56**	-0.58**	
**H**_6_	-0.32**	-0.53**	-0.59**	-0.49**	-0.26**	-0.29**	-0.51**	-0.59**	-0.51**	-0.36**	-0.55**	
	**MinT**_3_	**MinT**_4_	**MinT**_5_	**R**_1_	**R**_2_	**R**_3_	**R**_4_	**R**_5_	**H**_0_	**H**_1_	**H**_5_	**H**_6_
**MT**_1_												
**MT**_2_												
**MT**_3_												
**MT**_4_												
**MT**_5_												
**MaxT**_1_												
**MaxT**_2_												
**MaxT**_3_												
**MaxT**_4_												
**MinT**_1_												
**MinT**_2_												
**MinT**_3_	1.00											
**MinT**_4_	0.83**	1.00										
**MinT**_5_	0.47**	0.83**	1.00									
**R**_1_	0.47**	0.13	-0.26**	1.00								
**R**_2_	0.69**	0.47**	0.14*	0.49**	1.00							
**R**_3_	0.69**	0.68**	0.47**	0.25**	0.48**	1.00						
**R**_4_	0.55**	0.69**	0.68**	-0.04	0.26**	0.48**	1.00					
**R**_5_	0.23**	0.56**	0.69**	-0.29**	-0.03	0.26**	0.48**	1.00				
**H**_0_	0.56**	0.42**	0.17*	0.46**	0.48**	0.41**	0.23**	0.06	1.00			
**H**_1_	0.57**	0.56**	0.43**	0.47**	0.46**	0.48**	0.41**	0.24**	0.58**	1.00		
**H**_5_	-0.46**	-0.20**	0.11	-0.53**	-0.51**	-0.33**	-0.03	0.47**	-0.29**	-0.17*	1.00	
**H**_6_	-0.58**	-0.46**	-0.21**	-0.38**	-0.52**	-0.51**	-0.33**	-0.04	-0.33**	-0.29**	0.56**	1.00

*, *P* < 0.05.

**, *P* < 0.01

### Model construction

Our stepwise least square method results led us to select R_2_, MinT_0_, MinT_3_, N_1_, N_12_, and R_1_, R_2_, MT_1_, MaxT_1_, H_1_, N_1_, N_12_ as predictor variables in Model 1 for the 1971–1993 and 1994–2012 epochs, respectively. The expressions developed for Model 1 were as follows:

Model 1 (1971–1993 data):

$${\hat{y}}_{t}=1.707+5.022\times 10^{-3}{\text{R}}_{2}-2.529\times 10^{-2}{\text{MinT}}_{0}+0.127{\text{MinT}}_{3}+3.026\times 10^{-3}{\text{N}}_{1}+1.223\times 10^{-3}{\text{N}}_{12}$$

Model 1 (1994–2012 data):

$$\begin{array}{l} {\hat{y}}_{t}=-1.769-4.734\times 10^{-3}{\text{R}}_{1}+1.600\times 10^{-3}{\text{R}}_{2}+5.765\times 10^{-2}{\text{MT}}_{1}-7.609\times 10^{-3}{\text{MaxT}}_{1}\\ \;+4.784\times 10^{-2}{\text{H}}_{1}+1.398\times 10^{-2}{\text{N}}_{1}+1.251\times 10^{-2}{\text{N}}_{12} \end{array}$$

Based on our backfitting results, R_1_, R_3_, MT_1_, H_3_, N_1_, N_12_, and R_1_, R_2_, MT_1_, MaxT_2_, N_1_, N_12_ were selected as the predictor variables in Model 2 for the 1971–1993 and 1994–2012 epochs, respectively. The expressions developed for Model 2 were as follows:

Model 2 (1971–1993 data):

$${\hat{y}}_{t}=2.123+s({\text{R}}_{1})+s({\text{R}}_{3})+s({\text{MT}}_{1})+s({\text{H}}_{3})+s({\text{N}}_{1})+s({\text{N}}_{12})$$

Model 2 (1994–2012 data):

$${\hat{y}}_{t}=1.965+s({\text{R}}_{1})+s({\text{R}}_{2})+s({\text{MT}}_{1})+s({\text{MaxT}}_{2})+s({\text{N}}_{1})+s({\text{N}}_{12})$$

**Fig 3:**
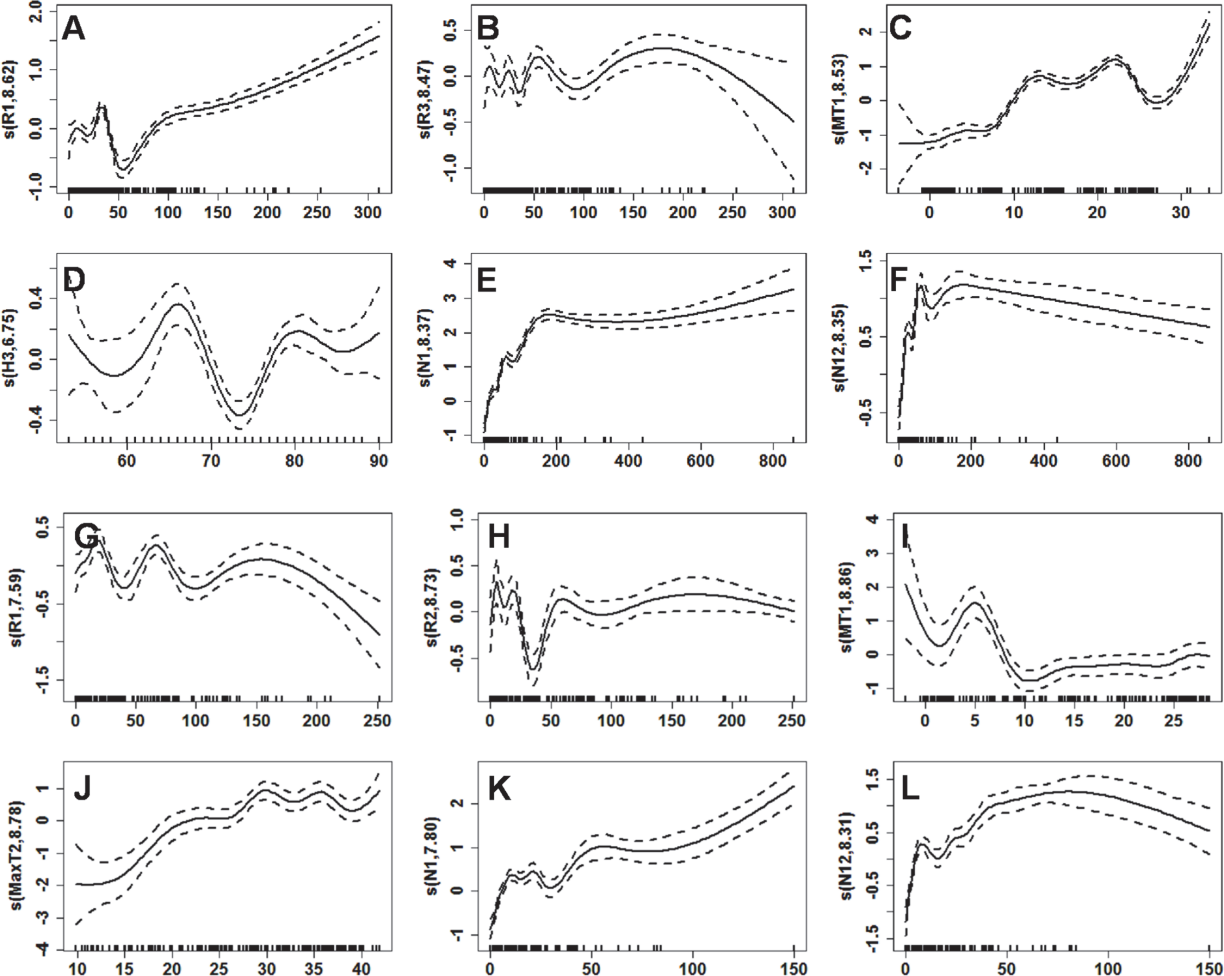
Curves of the smooth functions of each predictor variable in the GAM. (A–F) Curves of smooth functions of each predictor variable in the 1971–1993 epoch, including R_1_, R_3_, MT_1_, H_3_, N_1_, and N_12_. (G-L) Curves of smooth functions of each predictor variable in the 1994–2012 epoch, including R_1_, R_2_, MT_1_, MaxT_2_, N_1_, and N_12_. The x-axis shows the value of each predictor variable, and the y-axis shows the value of each smooth function. The dashed line is the estimated 95% confidence interval. The vertical lines adjacent to the lower x-axis show the presence of data in the matching years. The numbers in the labels of y-axis denote the effective degrees of freedom. These plots demonstrate the complex non-linear relationship between HFRS cases and predictor variables.

Plots of the smooth functions of each predictor variable in Model 2 are shown in Fig 3. Note that the curves show the complex non-linear relationship between the expected value of *y*_*t*_ and each predictor variable.

Three principal components were extracted from the preliminary predictor variables during the 1971–1993 epoch and two were extracted during the 1994–2012 epoch, with cumulative contribution rates of 83.47% and 78.81%, respectively. During the 1971–1993 epoch, component 1 represented MaxT_2_, MinT_1_, MinT_2_, MT_1_, and MT_2_, while component 2 represented MaxT_0_ and MT_0_, and component 3 represented H_3_. During the 1994–2012 epoch, component 1 represented MaxT_2_, MinT_2_, and MT_2_, while component 2 represented MaxT_1_ and H_1_. The expressions developed for Model 3 were as follows:

Model 3 (1971–1993 data):

$$y_{t}=7.030+0.644Z_{1}+0.284Z_{2}+0.069Z_{3}+0.342N_{1}+0.499N_{12}$$

Model 3 (1994–2012 data):

$$y_{t}=7.030+0.784Z_{1}+0.216Z_{2}+0.286N_{1}+0.487N_{12}$$

### Model evaluation

**Fig 4:**
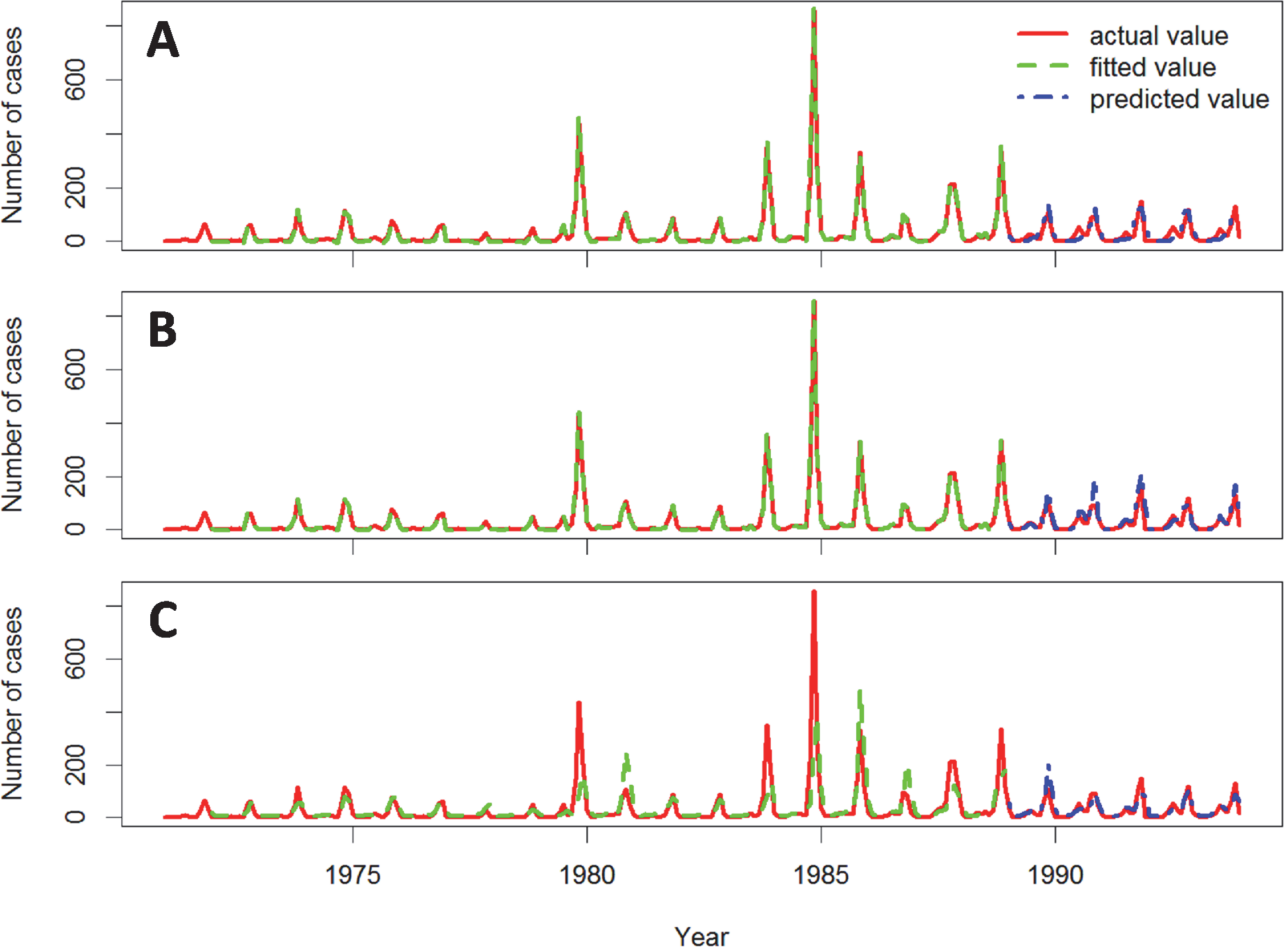
Actual, fitted, and predicted HFRS cases for the three models during the 1971–1993 epoch. Actual, fitted, and predicted HFRS cases curves for GLM (A), GAM (B), and PCRM (C).

**Fig 5:**
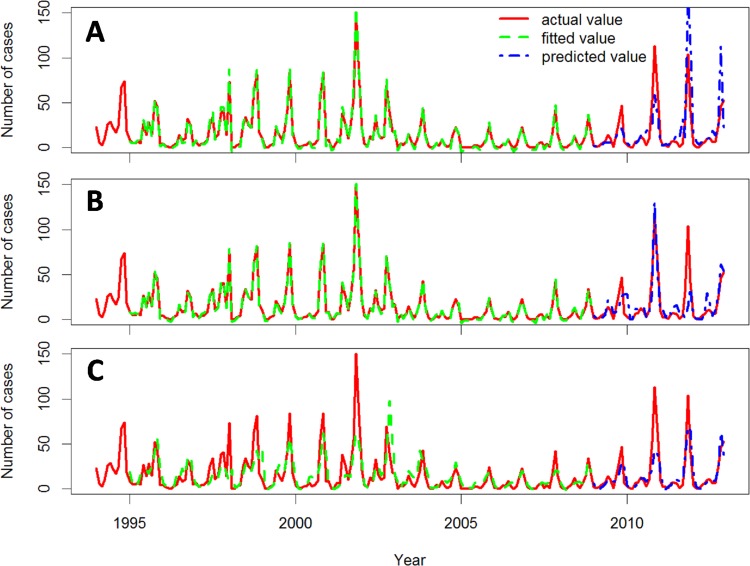
Actual, fitted, and predicted HFRS cases for the three models during the 1994–2012 epoch. Actual, fitted and predicted HFRS cases curves for GLM (A), GAM (B), and PCRM (C).

The actual, fitted, and predicted HFRS cases during the 1971–1993 and 1994–2012 epochs are showed in Figs 4 and 5, respectively. The autocorrelations of the fitting residuals of Model 2 in both epochs and those of Model 1 and 3 in the more recent epoch were not significant (Ljung-Box Q test, *P* > .05, Table 5), indicating that Model 2 in both epochs and Model 1 and 3 in the 1994–2012 epoch explain the association between the predictor variables and the incidence pattern for HFRS in Hu County over time. The autocorrelations of the predictive residuals of the three models were not significant for either time period (Ljung-Box Q test, *P* > .05, Table 5), indicating that the three models predicted the monthly numbers of HFRS cases accurately. The adjusted predictive *R* ^2^ values for Model 2 were higher than those for other two models in both epochs, indicating that Model 2 had the best predictive ability of the three models (Table 5). Therefore, Model 2 was accepted as the optimal model for HFRS prediction in Hu County. The adjusted *R* ^*2*^ values were lower in the early epoch than in the recent epoch for all three models.

**Table 5:** The adjusted fitting and predictive *R* ^2^, Ljung-Box *Q* statistics and *p* values of three models.

Model	1971–1993						1994–2012					
	Fitting			Predictive			Fitting			Predictive		
	adjusted *R* ^*2*^	*Q*	*P*	adjusted *R* ^*2*^	*Q*	*P*	adjusted *R* ^*2*^	*Q*	*P*	adjusted *R* ^*2*^	*Q*	*P*
**GLM**	0.997	25.690	4.001×10^–7^	0.752	0.670	0.413	0.984	1.480	0.224	0.669	2.424	0.120
**GAM**	0.959	0.099	0.753	0.799	3.712	0.054	0.818	0.258	0.611	0.756	2.358	0.125
**PCRM**	0.468	9.533	0.002	0.665	1.727	0.189	0.525	2.399	0.121	0.574	3.199	0.074

GLM: generalized linear model.

GAM: generalized additive model.

PCRM: principal component regression model.

*Q*: Ljung-Box *Q* statist.

## Discussion

Although HFRS epidemic prediction models have been constructed for many geographic areas [9, 10, 12], they are difficult to apply elsewhere, because the contingencies affecting HFRS outbreak levels vary regionally. For example, the southern oscillation was found to be a risk factor favoring larger HFRS outbreaks in Changsha [10] and Inner Mongolia [12], but a protective factor dampening HFRS outbreaks in Heilongjiang [9]. Although relative humidity was found to be a risk factor in both Heilongjiang [9] and Inner Mongolia [12], the correlation coefficients and parameters of the prediction models differed between these two areas. In the present study, we construct an HFRS prediction model optimized for Hu County based on the relationship between the local epidemiological history of HFRS cases before and after the implementation of vaccinations and concurrent meteorological factors. Comparing results obtained with a GLM, a GAM and a PCRM led us to conclude that the GAM had superior predictive ability relative to the GLM and the PCRM for both the pre- and post vaccination time periods. Redundancy cased by multi-collinearity between meteorological variables in the GLM was minimized by applying the stepwise least square method, while the backfitting method was used in the GAM, and principal components were extracted from the PCRM in the model-fitting process. Both the fitted and predicted residuals of the GAM were white noise in both time periods. Therefore, we conclude that a GAM can be used to predict HFRS trends in Hu County accurately using meteorological factors.

GAMs are flexible in that they allow non-parametric fits with relaxed assumptions on the relationship between response and predictor variables. This characteristic has made GAMs useful for studies examining the risk factors of respiratory diseases [23], coronary heart disease mortality [24], and autistic disorder [25], among other conditions. The GAM may be more robust than the GLM for interpreting relationships between response and predictor variables [17, 26, 27]. Our study provided additional evidence of this GAM advantage in the first application of these models to HFRS incidence prediction. The predictor variables included in our GAM for Hu County may not be maintained in GAMs for other locations with differing rodent host population, environmental characteristics and socio-economic factors. Nevertheless, the flexibility of the GAM in terms of setting predictor variables should be help yield high predictive accuracy.

The results of this study showed that the variables R_1_, R_2_, MT_1_ and MaxT_2_ were in non-linear relationship with monthly HFRS case numbers, and could be used to predict HFRS case numbers in Hu County in the 1994–2012 epoch. Consequently, epidemiologists should be able to use data describing ongoing fluctuations in these variables to predict future HFRS surges in Hu County accurately. Such predictions will enable targeted countermeasures to be prepared ahead of time and thus implemented promptly, which should improve the effectiveness of HFRS surveillance and control programs. This study provides practical evidence for the usefulness of the GAM in HFRS prediction, with the optimal predictive meteorological variables being determined for each particular locality.

It should be noted that the adjusted *R* ^2^ values of the three models in the post-vaccination time period were less than those in the preceding period, which indicates that the predictive power of all three models declined after 1994. Thus, there may be other factors that became more influential after the introduction of vaccines. It has been reported that increasing vaccination compliance plays an important role in reducing HFRS incidence in Hu County [8]. Therefore, we infer that the shift in the models’ predictive abilities after 1994 is related, at least in part, to the increasing vaccination compliance in Hu County. The vaccination factor was not included in our models as an explanatory variable because annual vaccination compliance data were tracked in Hu County after 1994, whereas monthly numbers of HFRS cases and meteorological data have been recorded consistently since 1971. It will be important to monitor the GAM’s predictive accuracy in Hu County in the coming years. If the adjusted *R* ^*2*^ values demonstrate further decreases, then some other methods should be pursued to adjust the model with respect to vaccination compliance data.

This study had a couple limitations which should be noted. Firstly, in the construction of our prediction model, we considered only meteorological factors and HFRS cases numbers in previous months. We did not account for other potential influencing factors such as rodent density, changes in land-use, and fluctuations in the non-immunized population, which may have decreased the predictive ability of the model. However, relative to data describing other environmental factors, meteorological data are highly objective, accurate, and contiguous, and are readily available. Thus a model constructed based on meteorological data will be more applicable than if it had been based on relatively subjective, inaccurate, discontinuous, or difficult to obtain data. Moreover, the goodness and stability of fit and the predictive capacity of the GAM in this study demonstrated that meteorological factors inform the HFRS epidemic pattern to a great extent and can be used to predict HFRS outbreaks accurately in Hu County. Secondly, the diagnostic criteria for HFRS changed in 1982. However, because the clinical diagnostic criteria did not change and the clinical symptoms of HFRS are easy to identify, most clinically-diagnosed HFRS cases were laboratory-confirmed after 1982. Therefore, we are not concerned that the diagnostic policy change of 1982 affects the suitability of the present GAM models.

In conclusion, this study demonstrated that the GAM is superior to the GLM and PCRM for HFRS case number prediction in Hu County. Additionally, our finding that the models’ predictive power shifted the same year that hantavirus vaccination programs were implemented in Hu County provides additional evidence of the importance of vaccination in HFRS control and prevention. This study provides practical evidence for further use of GAM in the epidemiological prediction of HFRS, especially within Hu County.

## Supporting Information

S1 Table The annual population in Hu County, China during 1971–2012. (DOCX)

## References

[1] Sargianou M, Watson DC, Chra P, Papa A, Starakis I, Gogos C, et al Hantavirus infections for the clinician: from case presentation to diagnosis and treatment. Crit Rev Microbiol. 2012;38: 317–329. 10.3109/1040841X.2012.673553 22553984

[2] Watson DC, Sargianou M, Papa A, Chra P, Starakis I, George P. Epidemiology of Hantavirus infections in humans: A comprehensive, global overview. Crit Rev Microbiol. 2014;40: 261–272. 10.3109/1040841X.2013.783555 23607444

[3] Huang LY, Zhou H, Yin WW, Wang Q, Sun H, Ding F, et al The current epidemic situation and surveillance regarding hemorrhagic fever with renal syndrome in China, 2010. Zhonghua Liu Xing Bing Xue Za Zhi. 2012;33: 685–691. 22968017

[4] Ministry of Health of China. 2012 China Health Statistical Yearbook. Beijing: Peking Union Medical College Press; 2012.

[5] Tan X, Xiao D, Yan Y. Analysis of epidemic situation of hemorrhagic fever with renal syndrome in Hu county, Xi'an, China from 1971 to 2010. Chin J Vector Biol & Control. 2012;23: 573–576. 16030375

[6] Xiao H, Gao LD, Li XJ, Lin XL, Dai XY, Zhu PJ, et al Environmental variability and the transmission of haemorrhagic fever with renal syndrome in Changsha, People's Republic of China. Epidemiol Infect. 2013;141: 1867–1875. 10.1017/S0950268812002555 23158456 PMC9151413

[7] Viel JF, Lefebvre A, Marianneau P, Joly D, Giraudoux P, Upegui E, et al Environmental risk factors for haemorrhagic fever with renal syndrome in a French new epidemic area. Epidemiol Infect. 2011;139: 867–874. 10.1017/S0950268810002062 20822577

[8] Xiao D, Wu K, Tan X, Yan T, Li H, Yan Y. The impact of the vaccination program for hemorrhagic fever with renal syndrome in Hu County, China. Vaccine. 2013;32: 740–745. 10.1016/j.vaccine.2013.11.024 24252696

[9] Li CP, Cui Z, Li SL, Magalhaes RJ, Wang BL, Zhang C, et al Association between Hemorrhagic Fever with Renal Syndrome epidemic and climate factors in Heilongjiang Province, China. Am J Trop Med Hyg. 2013;89: 1006–1012. 10.4269/ajtmh.12-0473 24019443 PMC3820312

[10] Xiao H, Tian HY, Cazelles B, Li XJ, Tong SL, Gao LD, et al Atmospheric moisture variability and transmission of hemorrhagic fever with renal syndrome in Changsha City, Mainland China, 1991–2010. PLOS Negl Trop Dis. 2013;7: e2260 10.1371/journal.pntd.0002260 23755316 PMC3674989

[11] Han SS, Kim S, Choi Y, Kim S, Kim YS. Air pollution and hemorrhagic fever with renal syndrome in South Korea: an ecological correlation study. BMC Public Health. 2013;13: 347 10.1186/1471-2458-13-347 23587219 PMC3641006

[12] Zhang WY, Guo WD, Fang LQ, Li CP, Bi P, Glass GE, et al Climate variability and hemorrhagic fever with renal syndrome transmission in Northeastern China. Environ Health Perspect. 2010;118: 915–920. 10.1289/ehp.0901504 20142167 PMC2920909

[13] Wang L, Liu Q. Bayesian Network Inference Based research on transmission mechanism of hemorrhagic fever with renal syndrom in China. Foreign Medical Sciences (Section of Medgeography). 2010;31: 216–220.

[14] Li Q, Guo NN, Han ZY, Zhang YB, Qi SX, Xu YG, et al Application of an autoregressive integrated moving average model for predicting the incidence of hemorrhagic fever with renal syndrome. Am J Trop Med Hyg. 2012;87: 364–370. 10.4269/ajtmh.2012.11-0472 22855772 PMC3414578

[15] Liu Q, Liu X, Jiang B, Yang W. Forecasting incidence of hemorrhagic fever with renal syndrome in China using ARIMA model. BMC Infect Dis. 2011;11: 218 10.1186/1471-2334-11-218 21838933 PMC3169483

[16] Lin H, Zhang Z, Lu L, Li X, Liu Q. Meteorological factors are associated with hemorrhagic fever with renal syndrome in Jiaonan County, China, 2006–2011. Int J Biometeorol. 2014;58: 1031–1037. 10.1007/s00484-013-0688-1 23793957

[17] Hu W, Mengersen K, Bi P, Tong S. Time-series analysis of the risk factors for haemorrhagic fever with renal syndrome: comparison of statistical models. Epidemiol Infect. 2007;135: 245–252. 16780612 10.1017/S0950268806006649 PMC2870560

[18] Liu X, Jiang B, Gu W, Liu Q. Temporal trend and climate factors of hemorrhagic fever with renal syndrome epidemic in Shenyang City, China. BMC Infect Dis. 2011;11: 331 10.1186/1471-2334-11-331 22133347 PMC3247297

[19] Hastie TJ, Tibshirani RJ. Generalized additive models London: Chapman and Hall; 1990.

[20] The central people's government of the people's republic of China website. The national kidney syndrome hemorrhagic fever monitoring programme. Available: http://www.gov.cn/yjgl/2005-09/10/content_30772.htm. Accessed 2014 Jun 15.

[21] Zhang L, Wilson DP. Trends in notifiable infectious diseases in China: implications for surveillance and population health policy. PLOS ONE. 2012;7: e31076 10.1371/journal.pone.0031076 22359565 PMC3281048

[22] Zhang YZ, Zou Y, Fu ZF, Plyusnin A. Hantavirus infections in humans and animals, China. Emerg Infect Dis. 2010;16: 1195–1203. 10.3201/eid1608.090470 20678311 PMC3298307

[23] Wanka ER, Bayerstadler A, Heumann C, Nowak D, Jorres RA, Fischer R. Weather and air pollutants have an impact on patients with respiratory diseases and breathing difficulties in Munich, Germany. Int J Biometeorol. 2013;58: 249–262. 10.1007/s00484-013-0730-3 24091656

[24] Wang DZ, Jiang GH, Zhang H, Song GD, Zhang Y. Effect of air pollution on coronary heart disease mortality in Tianjin, 2001–2009: a time-series study. Zhonghua Liu Xing Bing Xue Za Zhi. 2013;34: 478–483. 24016439

[25] Gunnes N, Suren P, Bresnahan M, Hornig M, Lie KK, Lipkin WI, et al Interpregnancy interval and risk of autistic disorder. Epidemiology. 2013;24: 906–912. 10.1097/01.ede.0000434435.52506.f5 24045716

[26] Knoblauch K, Maloney LT. Estimating classification images with generalized linear and additive models. J Vis. 2008;8: 10.1–10.19. 10.1167/8.16.10 PMC2861307 19146276

[27] Erbas B, Hyndman RJ. Sensitivity of the estimated air pollution-respiratory admissions relationship to statistical model choice. Int J Environ Health Res. 2005;15: 437–444. 16506437 10.1080/09603120500289192

