# Supplementary material for: Modeling and Predicting Hemorrhagic Fever with Renal Syndrome Trends Based on Meteorological Factors in Hu County, China
Source: PLoS One. 2015 Apr 13;10(4):e0123166. doi: 10.1371/journal.pone.0123166 (PMC4395290; doi:10.1371/journal.pone.0123166)
Supplement: S1 Table — (DOCX) [file pone.0123166.s001.docx]

**S1 Table. The annual population in Hu County, China during 1971-2012.**

| **year** | **population** |
| --- | --- |
| 1970 | 401600 |
| 1971 | 412700 |
| 1972 | 422600 |
| 1973 | 430200 |
| 1974 | 437200 |
| 1975 | 442800 |
| 1976 | 448900 |
| 1977 | 453900 |
| 1978 | 456900 |
| 1979 | 458270 |
| 1980 | 458546 |
| 1981 | 464169 |
| 1982 | 471543 |
| 1983 | 475681 |
| 1984 | 481831 |
| 1985 | 487762 |
| 1986 | 495628 |
| 1987 | 504834 |
| 1988 | 507333 |
| 1989 | 516130 |
| 1990 | 527953 |
| 1991 | 534404 |
| 1992 | 540104 |
| 1993 | 544656 |
| 1994 | 549264 |
| 1995 | 554867 |
| 1996 | 561591 |
| 1997 | 557524 |
| 1998 | 558810 |
| 1999 | 559881 |
| 2000 | 563038 |
| 2001 | 563248 |
| 2002 | 564705 |
| 2003 | 568625 |
| 2004 | 578065 |
| 2005 | 576845 |
| 2006 | 583078 |
| 2007 | 589614 |
| 2008 | 595081 |
| 2009 | 598483 |
| 2010 | 597071 |
| 2011 | 600675 |
| 2012 | 599442 |
